# Heterosis Increases Fertility, Fecundity, and Survival of Laboratory-Produced F_1_ Hybrid Males of the Malaria Mosquito *Anopheles coluzzii*

**DOI:** 10.1534/g3.115.021436

**Published:** 2015-10-20

**Authors:** Nkiru E. Ekechukwu, Rowida Baeshen, Sékou F. Traorè, Mamadou Coulibaly, Abdoulaye Diabate, Flaminia Catteruccia, Frédéric Tripet

**Affiliations:** * Centre for Applied Entomology and Parasitology, School of Life Sciences, Keele University, Staffordshire ST5 5BG, UK; † Malaria Research and Training Center, Faculty of Medicine and Dentistry, University of Mali, BP E.2528, Bamako, Mali; ‡ Institut de Recherche en Sciences de la Santé/Centre Muratz, 01 BP 390 Bobo-Dioulasso, Burkina Faso; § Harvard T.H. Chan School of Public Health, Boston, Massachusetts 02115; ** Dipartimento di Medicina Sperimentale e Scienze Biochimiche, Università degli Studi di Perugia, 06132 Perugia, Italy

**Keywords:** hybrid vigor, heterosis, inbreeding, colonization, mating competitiveness

## Abstract

The success of vector control strategies aiming to decrease disease transmission via the release of sterile or genetically-modified male mosquitoes critically depends on mating between laboratory-reared males and wild females. Unfortunately, mosquito colonization, laboratory rearing, and genetic manipulations can all negatively affect male competitiveness. Heterosis is commonly used to produce domestic animals with enhanced vigor and homogenous genetic background and could therefore potentially improve the mating performance of mass-reared male mosquitoes. Here, we produced enhanced hybrid males of the malaria mosquito *Anopheles coluzzii* by crossing two strains colonized >35 and 8 years ago. We compared the amount of sperm and mating plug proteins they transferred to females, as well as their insemination rate, reproductive success and longevity under various experimental conditions. Across experiments, widespread adaptations to laboratory mating were detected in the older strain. In large-group mating experiments, no overall hybrid advantage in insemination rates and the amount of sperm and accessory gland proteins transferred to females was detected. Despite higher sperm activity, hybrid males did not appear more fecund. However, individual-male mating and laboratory-swarm experiments revealed that hybrid males, while inseminating fewer females than older inbred males, were significantly more fertile, producing larger mating plugs and drastically increasing female fecundity. Heterotic males also showed increased longevity. These results validate the use of heterosis for creating hybrid males with improved fitness from long-established inbred laboratory strains. Therefore, this simple approach could facilitate disease control strategies based on male mosquito releases with important ultimate benefits to human health.

There is currently much interest in suppressing populations of the main malaria vectors in Africa via the release of radio-sterilized males and genetically-manipulated sterility-inducing males, or in replacing those populations through the deployment of genetically-modified mosquitoes unable to transmit malaria. These research efforts are fueled by the need for novel vector control approaches that can complement pesticide-based strategies that predictably fail over time due to the development of resistance in vector populations (Raghavendra *et al.* 2011; Hemingway 2014). As an example, the current spread of multiple resistance to insecticides observed in *Anopheles gambiae* s.l. populations across Africa (Ranson *et al.* 2011; Mitchell *et al.* 2012) could negate the achievements of mass-distribution of long-lasting insecticide-treated nets (LLINs) and large-scale indoor residual spraying (IRS) programs (Lengeler 2004; Pluess *et al.* 2010) in the foreseeable future (Sokhna *et al.* 2013). Modern mosquito release strategies not only offer new tools for vector control but can also complement current vaccine and drug treatment programs that are themselves vulnerable to the development of resistance by malaria parasites (Hyde 2005; Noedl 2013; White *et al.* 2014).

Whether they aim at population suppression via sterility-inducing males, or spreading genes to reduce malaria transmission (Alphey 2014; Burt 2014) genetically-engineered mosquito releases require the production of large numbers of males capable of successfully inseminating females from target populations (Benedict *et al.* 2009; Howell and Knols 2009). Mating competitiveness is truly the Achilles’ heel of mosquito release programs targeting malaria vectors in Africa because of the intricate structure of their populations: 1) *An. gambiae* s.l. is a complex of recently-diverged ’incipient species’ (Della Torre *et al.* 2002; Fontaine *et al.* 2015) characterized by large geographical ranges (Bayoh *et al.* 2001; Rogers *et al.* 2002); 2) these ranges commonly overlap such that several cryptic taxa often co-occur in otherwise similar ecological zones (Toure *et al.* 1998; Della Torre *et al.* 2002); 3) with the exception of limited hybrid zones (Caputo *et al.* 2011; Lee *et al.* 2013), cryptic taxa are also characterized by strong assortative mating (Tripet *et al.* 2001; Dabire *et al.* 2013); 4) therefore females from each subtaxa have evolved highly specific mate choice mechanisms. For all these reasons, producing large numbers of males with adequate mating preferences, thus making them competitive for targeting specific *An. gambiae* populations, will be a particularly complex and challenging endeavor (Diabate and Tripet 2015).

One of the major lessons learned from the discontinued 1970s sterile-male release programs is that genetic and environmental processes associated with colonization and long-term laboratory rearing negatively affect the reproductive phenotype of male anophelines (Asman *et al.* 1981; Benedict and Robinson 2003; Benedict *et al.* 2009; Howell and Knols 2009). In addition, laboratory studies carried out in different anopheline species have shown that genetic-modifications often carry a non-negligible fitness cost (Marrelli *et al.* 2006; Paton *et al.* 2013b; Catteruccia *et al.* 2003). Finally, semifield studies have shown that, independent of colonization and long-term laboratory maintenance effects, *An. gambiae* mate choice is affected by laboratory-rearing (Paton *et al.* 2013a). Taken together, this evidence suggests that the effects of colonization, laboratory rearing and genetic modifications have the potential to drastically decrease the proportion of released males effectively mating with wild females. Consequently, new schemes aiming specifically at improving the mating competitiveness of mass-reared anophelines are urgently needed.

Hybrid vigor, also known as heterosis, occurs when two inbred parental lines are crossed resulting in highly heterozygous offspring typically characterized by very few deleterious alleles at the homozygous state (Kristensen and Sorensen 2005). This outbreeding scheme is commonly used in domestic animals and plants breeding in order to produce offspring with enhanced genetic and phenotypic quality and homogenous genetic background (Kristensen and Sorensen 2005). Surprisingly, the full potential of heterosis has been exploited only rarely in sterile insect release programs, where hybridization or outcrossing of old laboratory strains with younger strains or wild individuals has often been used for limiting problems associated with selection for insectary-maintenance, genetic drift and inbreeding (Shelly 2001; Rull and Barreda-Landa 2007; McInnis *et al.* 2002), but only rarely for generating F_1_ progeny with enhanced mating competitiveness from inbred established lines for immediate release (Gilchrist and Meats 2014; Bellini *et al.* 2013). In Aedines, the technique was tested in the 1970s on inbred colonized strains *Aedes aegypti* males and resulted in an overall improvement of male competitiveness (Seawright *et al.* 1977). However, in a recent study focusing on radiated males *Ae. albopictus* from more-recently colonized strains, no clear hybrid advantage was detected (Bellini *et al.* 2013).

The application of heterosis for the production of anopheline mosquitoes with improved mating competitiveness has recently been proposed and could be particularly rewarding given the low success of past release programs (Benedict and Robinson 2003). In a comparative study, Baeshen *et al.* (2014) showed that inbreeding and selection for laboratory rearing conditions led to fast changes in the male reproductive phenotype of colonized mosquitoes (Baeshen *et al.* 2014). Within 2 years of colonization, inbreeding was found to significantly affect sperm length, which decreased by as much as ∼2.5-fold in inbred strains over 35 years old (Baeshen *et al.* 2014). Critically, heterosis restored sperm length in the male progeny of crosses between such old inbred strains, suggesting that this simple genetic process could be used to create enhanced hybrid males with generally improved reproductive phenotypes (Baeshen *et al.* 2014). Heterosis could improve male competitiveness in multiple ways. First, because mating in the anophelines occurs in swarms, and swarming is a particularly energy costly process (Maiga *et al.* 2014), enhanced male vigor may increase their ability to effectively compete with wild males for access to females. Second, if vigor is a phenotypic cue used by females in choosing their mates, heterosis could further increase the mating success of released males by making them more attractive to females. During mating, anopheline males transfer sperm to females as well as a mating plug whose matrix is constituted of *Plugin* protein and TG3, transglutaminase enzyme (Rogers *et al.* 2009; Le *et al.* 2013) and which is loaded with sex-peptides and hormones that are crucial to a number of reproductive traits, including fecundity, sperm storage, fertility, oviposition and susceptibility to further mating (Pondeville *et al.* 2008; Rogers *et al.* 2009; Baldini *et al.* 2013; Shaw *et al.* 2014; Gabrieli *et al.* 2014). Therefore, heterosis could boost the overall sexual performance of hybrid males by increasing the total number of females they can successfully inseminate and transfer mating plugs to. Finally, heterosis could also enable hybrid males to transfer better quality sperm and mating plugs to each these females, thereby affecting their fecundity and fertility.

Here, we conducted a series of laboratory experiments aiming at determining the mating rate, investment in sperm and mating plug, and reproductive success of hybrid males produced by crossing two inbred *An. coluzzii* strains. Large-group mating experiments were complemented with individual-male mating experiments and analyses of couples captured in copula from laboratory swarm experiments. Finally, the longevities of inbred and hybrid males were assessed under standard insectary conditions and strong hydric stress. In addition to revealing reproductive traits negatively affected by inbreeding and thus enhanced by heterosis, the experiments allowed us to identify profound changes in the male reproductive phenotype resulting from adaptations to mating in the laboratory. Importantly, the results suggest that the simple principle of heterosis can be used to drastically improve male mosquito fitness and thus the effectiveness mosquito-release programs aimed at decreasing or interrupting malaria transmission in areas of Africa endemic for the disease.

## Materials and Methods

### General mosquito husbandry

All experiments were conducted from 2009 to 2013 in dedicated insectaries of the Centre for Applied Entomology and Parasitology, Keele University, UK. Mosquito strains were kept at 27 ± 2°, 70 ± 5% relative humidity, with a 12-hr light/dark cycle. Larvae were grown at a density of 200 larvae/l and fed an optimized diet of ground fish food (Tetramin, Tetra, Melle, Germany) (Aboagye-Antwi and Tripet 2010). Upon pupation, pupae were transferred to a standard rearing cage made of a 5L white polypropylene bucket (∼20.5 cm height × 20 cm diameter) with a sleeved side opening for introducing and removing mosquitoes and accessories, and the top covered with mosquito netting. Adults were typically maintained at densities of 600–800 adults per enclosure and provided with water and a 5% glucose solution *ad libitum*. Females were bloodfed using horse blood provided via a Hemotek membrane feeding system (Discovery Workshops, Accrington, UK)

### Mosquito strains and field captures

Two mosquito lines with contrasted age since colonization were used. The ‘Mopti 2003’ strain is an 8-year-old wild-type strain of *An. coluzzii* colonized from the village of N’Gabakoro Droit in Mali, West Africa in 2003 (Baeshen *et al.* 2014). The Mopti strain is of the so-called Mopti chromosomal form, a West African population characterized by the *bc* and *u* inversion on chromosome 2R and fixed for inversion *a* on 2L (Toure *et al.* 1994, 1998). The KIL strain is a >35-year-old strain thought to have been colonized from the Marangu area in Tanzania in the 1970s (Gale and Crampton 1987). The KIL strain is of standard karyotypic arrangement on 2R and polymorphic for inversion *a* on 2L. Both strains are of the M molecular form at the diagnostic rDNA locus used to distinguish *An. coluzzii* from other sibling species (Coetzee *et al.* 2013). Given its East African origin, it is suspected that the KIL strain’s origin has been mistaken, or that, what was originally an S molecular form strain, became historically contaminated by *An. coluzzii* strain. While maintained in our insectaries the strains were kept in separate insectaries under otherwise identical temperature, humidity and light/dark settings. For experiments, the progeny from these two strains, hybrid progeny from these two strains (see below), or the progeny from field-caught females were all reared from the first instar larval stage in the same insectary in order to avoid possible confounding factors due to subtle variations between insectaries.

Previous studies have shown that the sperm size of the KIL and Mopti 2003 is much reduced compared to that of more recently colonized strains as a result of inbreeding (Baeshen *et al.* 2014). The quality of reproductive traits that are negatively affected by inbreeding in mosquito colonies is expected to be restored in heterozygous males (Baeshen *et al.* 2014). Consequently, we created enhanced hybrid males by crossing 200 virgin females from the KIL strain with 200 virgin males from Mopti 2003 strain. In previous experiments hybrid males were characterized by sperm length comparable to that of male progeny from field-collected females (Baeshen *et al.* 2014).

In some cases, the male F_1_ progeny of wild-caught Mopti chromosomal form *An. coluzzii* females was used for comparison with heterotic and inbred males. To do so, blood-fed females were collected from huts in the same collection site used to create the Mopti 2003 strain and brought to the insectary at the Malaria Research and Training Centre, Bamako, Mali. Once gravid, females were placed in individual tubes for oviposition. Two days later, individual egg batches and female carcasses were shipped to Keele University. DNA extractions from females were carried out immediately using DNAzol (Invitrogen, Carlsbad, CA). The diagnostic PCR/RFLP protocol developed by Fanello *et al.* (2002) was used to differentiate *An. coluzzii* and *An. gambiae* s.s. (previously known as M and S molecular forms) females from those belonging the sister species *An. arabiensis*. Once successfully genotyped, the freshly hatched *An. coluzzii* broods (first instar larvae) were reared using a standard larval rearing protocol (see above). Adults were maintained under the same conditions as the other strains.

Adults in copula were collected in the Vallée du Kou (11°39′N, 04°41′W) a rice cultivating area ∼30 km North West of Bobo-Dioulasso, Burkina Faso from swarms molecularly characterized as the Mopti chromosomal form of *An. coluzzii*. Mating pairs were caught with sweep-nets as they were leaving swarms flying in tandem. They were blown into small individual netted cups to complete mating and brought back to the Institut de Recherche des Sciences de la Santé in Bobo Dioulasso. Mating pairs were then transferred to 1.5 ml centrifuge tubes and stored at –20° before being shipped chilled to Keele University.

### Estimates of genetic diversity in field population and laboratory strains

The mean expected heterozygosity or genetic diversity within population, *Hs* (Nei 1987), was estimated for the field Mopti population from Burkina Faso from which field individuals were collected, the Mopti 2003 and the KIL laboratory colonies using single nucleotide polymorphisms (SNP) identified by resequencing of DNA pools.

#### Genome sequencing of laboratory strains:

Archived DNA from 13 Mopti 2003 and 17 KIL female mosquitoes stored in 2009 at the beginning of the study was amplified by multiple-displacement amplification using the Illustra GenomiPhiV2 DNA Amplification kit (GE Healthcare Bio-sciences, Piscataway, NJ), purified using a MinElute Reaction Cleanup Kit (Qiagen, Hilden, Germany) and DNA pools were sent to the Liverpool Centre for Genomic Research (CGR, Liverpool, UK) for sequencing, mapping and variants detection. DNA libraries were prepared according to the Illumina TruSeq DNA protocol (Illumina, San Diego, CA), multiplexed and sequenced on two lanes of an Illumina HiSequation 2000 sequencer.

Base-calling of indexed reads was performed with the program CASAVA 1.8.2 (Illumina). The reads were trimmed using the software Cutadapt 1.2.1 (Martin 2011) and Sickle 1.200 (Joshi and Fass 2011) and mapped to the *An. gambiae* (PEST) reference sequence (assembly AgamP3) using Bowtie 2.1.0 (Langmead and Salzberg 2012). Alignments were filtered to remove low mapping quality reads and redundant duplicate reads were filtered out using the Picard MarkDuplicates Tool 1.85 (http://broadinstitute.github.io/picard). Mapped reads were locally re‐aligned around indels using the Genome Analysis Tool Kit (GATK) version 2.1.13 (McKenna *et al.* 2010; DePristo *et al.* 2011). The mean coverage depth after local realignment and duplicate removal of reads was equal to 34.1× for the Mopti parental strain and 26.2× for the KIL strain. Variant detection was performed using the GATK ‘UnifiedGenotyper’ package (McKenna *et al.* 2010; DePristo *et al.* 2011) with an expected SNP heterozygosity of 0.01. An expected ploidy of 20 was used (*i.e.*, allele frequencies calculated in increments of 5%) in order to best balance accurate sample representation and computational efficiency. Variants were further filtered using the GATK ‘VariantFiltration’ package (McKenna *et al.* 2010; DePristo *et al.* 2011).

#### Low coverage genome sequencing of Mopti field populations:

The DNA extractions from 30 field-collected female *An. gambiae* s.s. characterized as the Mopti form of *An. coluzzii* as described above were combined into a single pool that was purified using a MinElute Reaction Cleanup Kit (Qiagen) and sent to the CGR for sequencing, mapping and variants detection as described above. All procedures were as described for whole-genome sequencing of the Mopti and KIL strains with the exception that ∼5× coverage resequencing was conducted for this sample.

#### Calculation of multilocus heterozygosity:

Mean multilocus heterozygosities were calculated for each population based on SNPs identified in all three populations, satisfying GATK’s most stringent ’pass’ criteria with variant quality score ≥50 (Phred scale). We used solely SNPs located on chromosome 3 (Supporting Information, File S1) to avoid possible bias due to inversion polymorphisms present on chromosome 2 and to hemizygosity of the X chromosome (Slotman *et al.* 2007). Heterozygosity or gene diversity within the population was calculated using the formula:

$$H_{s}=1-\Sigma pi^{2}$$

where *p* and *i* are the mean frequencies of the major and minor alleles calculated in any given population (Nei 1987).

### Group mating experiments

#### Precopulatory sex-peptides in virgin males:

Prior to the group mating experiments, the amount of the *Plugin* and *transglutaminase* accessory-gland proteins, two major constituents of the mating plug transferred by males to females alongside sperm (Rogers *et al.* 2009), was compared in 4-day-old virgin hybrid males, males from the KIL and Mopti 2003 parental strains and in male progeny from field-collected females.

#### Experimental design:

Quantitative comparisons of transferred plugs and sperm were made between four experimental mating combinations of males and females. Hybrid males were mated to females from the KIL and Mopti 2003 females (two combinations) and KIL and Mopti 2003 males were mated with their own females (two combinations). For each mating combinations 200 2- to 4-day-old virgin males were combined with 200 2- to 4-day-old virgin females in a standard 5-L rearing cage. The experiment was made in three replicates using independent mosquito cohorts. Mating cages were set up between 3 and 5 pm to give the mosquitoes time to recover for swarming and mating at artificial nighttime (7 pm). The next morning (8 am), a random sample of 48 females from each cage was dissected for sex-peptide quantification (see below). Two other random samples of 24 females were taken, one for sperm vigor quantification, the other for sperm number quantification (see below). The remaining females were kept for 24 hr before being fed on horse blood using a Hemotek membrane feeding system (Discovery Workshops) for measurements of their fecundity (see below).

#### Quantification of sex peptides:

Fresh samples of 4-day-old virgin males and 3- to 5-day-old females mated overnight were chilled and dissected in a drop of PBS solution. Proteins were quantified using enzyme-linked immuno-sorbent assays (ELISA) following Wigby *et al.* (2009) with modifications. The last two mosquito abdominal segments were placed into microcentrifuge tubes containing 220 µl of proteinase inhibitor (PI) (Roche, Basel, Switzerland) and ground for 30 sec with a pestle then sonicated for 10 min using a Bioruptor sonicator (Diagenode, Denville, NJ). The samples were spun in a cold centrifuge and the supernatant collected for quantification by ELISA using affinity-purified polyclonal antibodies against the transglutaminase TG3 (AGAP009099), which is important for mating plug formation, and the major mating plug protein Plugin (AGAP009368) (Rogers *et al.* 2009; Le *et al.* 2013). All plate preparation steps followed standard ELISA procedures (Wigby *et al.* 2009). For absorbance readings, 100 µL of HRP substrate (TMB liquid substrate system, Sigma-Aldrich, St Louis, MO) was added to each well and the plates incubated in the dark for 10 min for Plugin and between 20 and 40 min for TG3. Reactions were stopped by adding 100 µl stop solution (9% H_3_PO_4_) and read at 450 nm using a Labsystems Multiskan plate reader (Thermofisher Scientific, Waltham, MA).

Standard curves were made using a pool of lower abdominal reproductive tracts from ten males of the Mopti 2003 strain using the same procedures as other samples. Two independent standard curves based on six two-fold dilutions were made for each plate. Based on these standard curves, all Plugin and TG3 sample readings were translated into ’male accessory gland equivalents’ or the average amount of each protein found in one accessory gland in Mopti 2003 males. All curve fittings and data transformations were conducted using the software JMP 10 (SAS Institute Inc., Cary, NC)

#### Quantification of sperm activity and spermathecae size:

Following overnight group mating, a random subsample of 24 female per cage were separated from males and transferred to a new cage. Sperm activation is thought to occur 16–17 hr post mating (Rogers *et al.* 2009), hence females were kept for 24 hr prior to measuring sperm activity. In this study, the sperm content of ∼45% of females was found to be still inactive 24 hr after mating. The proportion of females with inactive sperm did not differ significantly among experimental groups (Chi-square: *χ*^2^ = 4.21, df = 3, *n* = 192, *P* = 0.240). Subsequent analyses were conducted on females in which sperm activation had occurred by 24 hr.

Sperm activity quantifications were made through video recordings of sperm cells swirling inside the spermathecae. This technique was preferred over measures of motility or live/dead assays because these techniques are notoriously biased by the non-natural-like pH and solution composition in which the sperm is observed (Holman 2009) and optimal conditions for anopheline sperm are unknown. Females were briefly knocked down on ice and swiftly dissected in PBS buffer under a binocular microscope. Using a pair of microneedles, the last abdominal segment of the mosquito was opened and the intact spermathecae separated from the abdomen. Recordings were made by promptly transferring the microscope slides under an inverted phase-contrast microscope equipped with a digital video camera (Motic China Group Co Ltd, Xiamen, China) with light shining through the spermathecal wall and 200× magnification. The video recordings were visualized at a later stage and the sperm movement scored every 10 sec during the 1 min of recording and ranked as 1 (very low), 2 (low), 3 (medium), and 4 (high). Scoring of sperm activity was done ’blindly’ and by trained observers, a method that led to high repeatability in preliminary experiments. The median of the sperm vigor score was used for statistical comparisons.

The size of spermathecae was also measured from digital still pictures created from the digital video recordings. The perimeter of the spermathecae was drawn on screen using the software ImageJ 1.44 available at http://rsb.info.nih.gov/ij/download.html and their surface area calculated by the same program.

#### Sperm quantification:

Following the video recordings of sperm activity, each spermathecae was gently picked up with a dissecting pin and placed into a 1.5 ml centrifuge tube containing 500 µl of lysis buffer, and 10 µl of proteinase K. The mix was incubated overnight on a heat block set at 55°. The next day, DNA extraction was conducted using a ChargeSwitch gDNA micro tissue kit (Invitrogen) following the manufacturer’s instructions.

The number of sperm cells contained in the gDNA extracts from the spermathecae was estimated using a Taqman assay (Applied Biosystems, Foster City, CA) targeting a known Y-chromosome-specific sequence (Krzywinski *et al.* 2004). The forward primer was: TTACCACGCTGGCAAATGC; reverse primer: CGTGCAACAGCTCGTGATG; and probe: ACGCCGCATCCATCT. Quantitative real-time PCRs were run using TaqMan Universal Master Mix (Applied Biosystems) on a Step-One-Plus Real Time PCR System (Applied Biosystems). PCR steps were: 50° for 2 min, 95° for 10 min, 95° for 0.15 sec, and 60° for 1 min.

Threshold cycle values (CT) were translated into number of Y-chromosome copies using the standard curve method and a pool of male mosquito DNA as standard. The concentration of the pool of male DNA was first adjusted to 50,000 haploid genome copies/μl using a theoretical approximate molecular weight of 0.3 pg per genome, *i.e.*, 1.96e-21 g/bp × 278,253,050 bp (Holt *et al.* 2002). Two independent five-step serial dilutions curves were made per PCR plate. The curve with the best fit was used to translate CT-values into estimated numbers of Y-chromosomes/μl, which were multiplied by two to account for female sperm (assuming a 1:1 male to female sperm ratio) and multiplied by the total volume of the DNA extraction from inseminated spermathecae (75 μl). Spermathecae DNA extracts that did not lead to any PCR amplification after repeated attempts were considered as noninseminated and recorded as such for quantification of insemination rates.

Estimates of sperm numbers calculated from standard curves based on DNA extracted using the exact same conditions as the sperm DNA samples can be considered ’qualitative’. Technical replication also showed that the measurements were highly repeatable (Pearson correlation: *r* > 0.9 and *P* < 0.001 in most cases). Thus in the group mating experiments (this section) and individual male mating experiments (next section) sperm number estimates allowed for qualitative comparisons between groups.

#### Estimation of female fecundity:

Two days post blood feeding, 48 females were collected from the cage of each experimental group and set in individual tubes lined with a strip of filter paper for oviposition. After 2 days, all eggs were counted and were transferred to individual plastic trays containing 200 ml of distilled water to which a drop of Liquifry baby fish food (Interpet Ltd, Dorking, UK) was added. After 4 days to allow for egg hatching, all larvae were counted.

### Individual male mating experiments

#### Experimental design:

The four mating combinations used for large-group mating experiment were used for comparisons of individual male insemination rates, sperm transfer, and fecundity. Instead of combining large numbers of 2- to 4-day-old virgin males and females into large cages as above, an elaborate cage design was used to create conditions allowing single males to mate with several females. First, 20 males from each treatment group were placed in a small 120 ml polystyrene cup, covered with double mesh, and topped with a cotton wool pad soaked in 5% glucose solution. This cup was placed into a bigger cup (700 ml) containing 20 females from each treatment group and served to create swarm-like male flight-tones designed to stimulate mating by females with the single male accessible to them. The cages were closed with mesh and supplied with 5% glucose solution, and set-up from 3–5 pm in order to give captive ’swarming males’ and females time to time to recover for mating at artificial night time (7 pm). At around 6 pm, a single male from each treatment group was introduced into the larger cage with their group of females and kept with them for three successive nights. Twenty individual male mating cages were set-up for each experimental treatment. To minimize confounding factors, the shelf position of mating cages in the insectary was initially fully randomized among groups and their position shifted among shelves once per day.

After three nights of mating, individual males and the cup of ’swarming males’ were removed. Individual males were stored in ethanol 70% and female groups were transferred into a new 700 ml meshed cup equipped with an empty inverted oviposition cup and blood fed using an artificial membrane feeder. Two days later, the oviposition cup was inverted and filled with water. After a further 2 days, all females were collected and stored in ethanol 70% for ulterior dissections. The number of eggs and larvae per female group for each individual male mating cage were recorded as described previously.

#### Assessment of insemination status and sperm DNA extractions:

Females from all individual-male mating cages stored in ethanol 70% were dissected to determine the mating status and to extract sperm bundles as in previous studies (Tripet *et al.* 2001, 2005b). The sperm bundles were transferred to 500 ml of lysis buffer for overnight incubation and subsequent DNA extraction and sperm number quantification as described in the previous section.

#### Male and female body size measurement:

The wing length of individual males and all mated and unmated females was measured as an accurate correlate of their body size (Lyimo and Koella 1992). Wings were measured from the alular notch to the distal wing margin, excluding the fringe scales, to the nearest 0.01 mm using a binocular microscope with an eyepiece graticule.

### Mating plug and sperm transfer in experimental swarms

#### Experimental design:

The four mating combinations used for large-group and individual-male mating experiments were also used for comparisons of sperm transfer and plug transfer in experimental swarm conditions. Mating cages were prepared in the evening. One hundred 2- to 4-day-old virgin females were introduced into a standard 5L rearing cage in which 250 same-aged virgin males had been placed and given 6–8 hr to rest and feed on glucose solution. Dimmed sunset-like conditions were created by having diffuse light coming through a doorframe situated ∼4 m away from the cages. The cages themselves were placed on a 1.2 m high table. Swarming started usually 1–2 min after introducing females to the male cages and couples in copula dropping on the cage floor were immediately captured using a mouth aspirator and gently blown in individual tubes (50 ml) to complete mating. Couples were collected from cages for each treatment group sequentially for 30–60 min. A few minutes after they completed mating, couples were placed on ice and females transferred to 200 µl PBS buffer with proteinase inhibitor for storage at –20°, dissections at a later stage and body size measurement. Males were stored in ethanol 70% for size measurement at a later stage. In order to avoid potential bias due to the timing of swarming, experimental mating cages were set-up with four successive independent mosquito cohorts, each time alternating the sequence in which the swarms were initiated for each treatment groups.

#### Plug dissection and spermathecae dissections:

Females were thawed on ice prior to being dissected in a drop of PBS buffer under a Leica EZ4 binocular dissecting microscope (Leica Microsystems GmbH, Solms, Germany). Under the light spectrum produced by the dissecting scope’s integrated lighting system, the mating plug lodged in the female’s abdomen fluoresces thereby facilitating the dissection. The mating plug and surrounding reproductive tissues including the spermathecae were extracted by gently pushing downward on the female’s abdomen starting from the third segment and holding the rest of the body firmly with dissecting tools. With great care, the plugs and spermathecae were separated from surrounding tissues, the slide transferred under an inverted phase-contrast microscope equipped with a digital camera (Motic China Group Co Ltd.) and a still picture of the plug taken for measurement on screen using the software ImageJ as previously described for spermathecae measurements. Next, the slide was returned to the dissecting scope and the spermathecae collected and transferred to a 1.5 ml centrifuge in 500 ml of lysis buffer for overnight incubation and subsequent DNA extraction and sperm number quantification as described in the previous sections. In this instance, because the conditions used to store female bodies and the spermathecae differed from that of the male bodies used to extract DNA for the standard curves, sperm estimates could only be used for quantitative comparisons among groups and are presented as indices to avoid confusion. In a few instances, females were observed to have two plugs, either from the same male or from mating twice in quick succession. Because sperm quantification data from these females could not be related to the captured males with certainty, these were excluded from all analyses.

### Male survival experiments

Two hundred and forty 2- to 4-day-old male mosquitoes from the KIL and Mopti 2003 strains as well as hybrid males (see above) and the male progeny of field-captured females were randomly picked from the pupal emergence cages and distributed into 24 meshed cups (200 ml; 20 males per cups). The 24 cups (six per treatment groups) were assigned to two environmental conditions. The first group of 12 cages was kept under standard insectary conditions (see above) with water and sugar available at all times. These conditions were designed to reveal intrinsic male longevity independent of major environmental stressors. The cages were organized using a fully randomized design and their position shuffled daily in order to eliminate confounding environmental effects in the insectary. All cages were examined daily for dead mosquitoes, which were removed and stored individually in ethanol 70%.

The second group of 12 cups was placed in an incubator set at 30° and 30% relative humidity (RH) without water and sugar until death. These conditions are referred to as ‘intense desiccation’ throughout the text and were used here to simulate a life-threatening desiccation episode in nature. This methodology mirrors that used in physiological studies to reveal underlying differences in metabolic reserves (Mogi *et al.* 1996; Gray and Bradley 2006) and applied to previous physio-ecological studies of *An. gambiae* (Aboagye-Antwi and Tripet 2010; Aboagye-Antwi *et al.* 2010). Here it was used to reveal a possible hybrid advantage under harsh environmental conditions.

The cages were organized using a fully randomized design and their position shuffled regularly in the incubator in order to avoid confounding factors. All cages were examined every 3 hr for dead mosquitoes, which were removed and stored individually in ethanol 70%. Mosquitoes we considered ‘dead’ when they were too weak to fly and could not stand on their legs. Typically, these signs indicate that death will occur within the following hour. The body size of all males was later measured by measuring their wing length (see above).

### Statistical analyses

All statistical analyses were performed using the software JMP 10 (SAS Institute, Inc.). All data were checked for normality and heteroscedasticity and analyzed accordingly using parametric or nonparametric procedures. Direct and interactive replicate effects were always tested but are reported only when significant. Interactions between independent variables in multivariate analyses were always tested but removed from models in a step-wise manner when nonsignificant.

### Data availability

File S1 contains SNP allelic frequencies and heterozygosities for KIL, Mopti, and field Mopti populations.

## Results

### Genetic diversity in field population and laboratory colonies

A total of 80,165 di-allelic SNPs were identified on chromosome 3 and used to estimate the multilocus heterozygosity *Hs* of the field Mopti population from Burkina Faso, the >8-year-old Mopti 2003 laboratory strain and the >35-year-old KIL strain (File S1). Gene diversity significantly decreased from 0.156 (95% CIs 0.155–0.157) in the field Mopti population, to 0.126 (0.126–0.127) in the Mopti 2003 strain, and 0.122 (0.120–0.123) in the older KIL strain supporting the idea of an increase in inbreeding in relation to colonization and laboratory rearing (Wilcoxon signed-rank test: *P* < 0.001 in all cases).

### Group mating experiments

#### Precopulatory Plugin and TG3 levels in virgin males:

**Figure 1:**
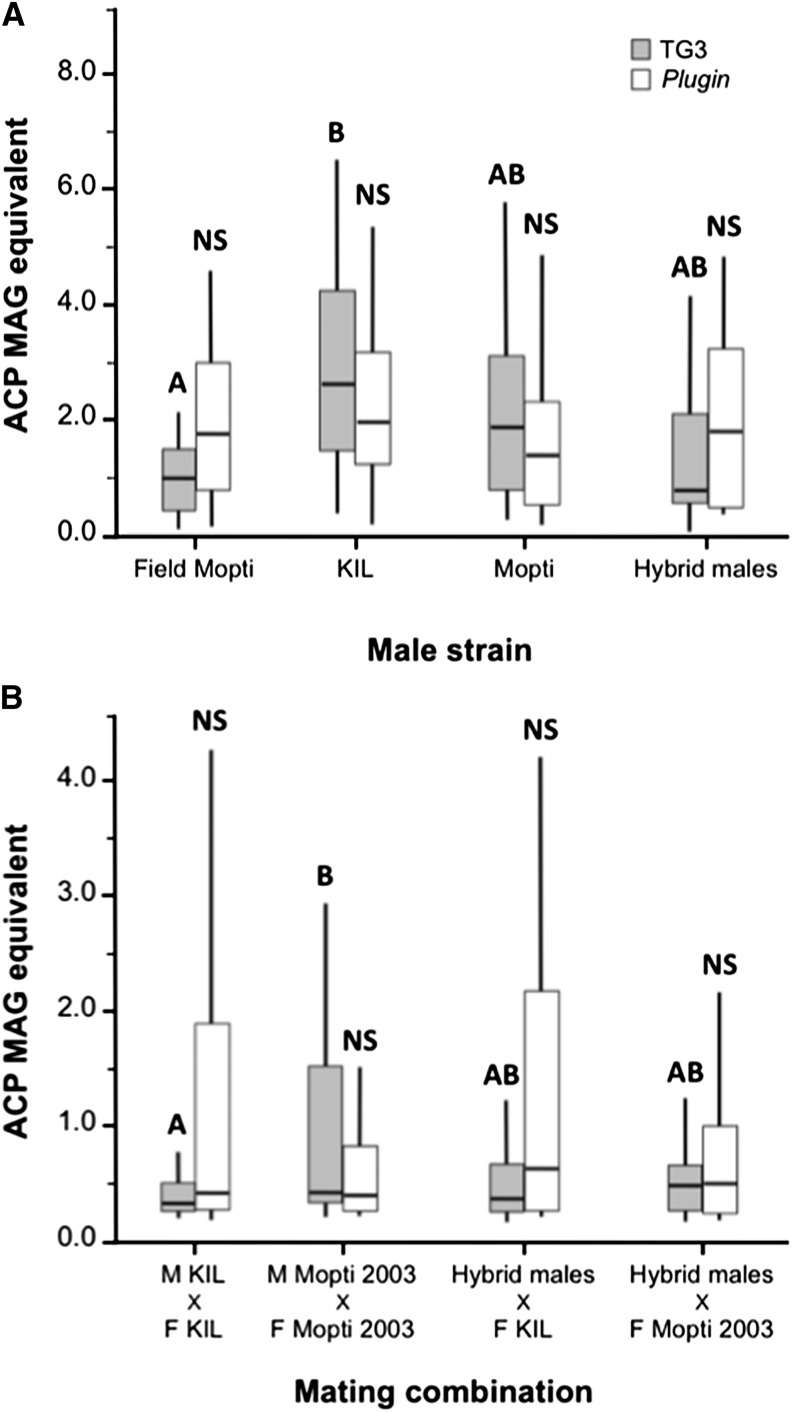
Male mating plug protein production and transfer to females in large-group mating cage experiments. (A) Precopulatory levels of TG3 (gray bars) and *Plugin* (white bars) were quantified by ELISA in virgin male progeny from field-caught females, males of the KIL and Mopti strains and in heterotic males. (B) The mating plug proteins were also quantified in KIL and Mopti females mated to KIL and Mopti males or hybrid males. Boxplots indicate medians and first and third quartiles and whiskers are the same quartiles ±(1.5 × interquartile range). Bars sharing a letter are nonsignificantly different, those with different letters significantly different.

The amount of TG3, the transglutaminase enzyme necessary for plug formation (Rogers *et al.* 2009; Le *et al.* 2013) and *Plugin*, the major mating plug protein (Rogers *et al.* 2009) in the accessory glands of 7-day-old virgin males was quantified in the KIL, Mopti strains, in hybrid males as well as in the male progeny of field-caught females (Figure 1A). TG3 was found to be significantly more abundant in the male accessory glands (MAGS) of the long-established KIL strain than in male field progeny (Kruskal-Wallis: *χ*^2^ = 27.7, df = 3, *n* = 145, *P* < 0.001; Dunn’s pairwise comparison: *Z* = 4.10, *n* = 60, *P* < 0.001). Mopti males and hybrid males had intermediate levels of TG3 and did not differ significantly from the previous two groups (Dunn’s comparisons: *P* > 0.098 in all cases) (Figure 1A). There were no significant differences in the amount of Plugin measured from precopulatory accessory glands of field, KIL, Mopti, and heterotic males (Kruskal-Wallis: *χ*^2^ = 3.87, df = 3, *n* = 142, *P* = 0.275).

#### Plugin and TG3 transfer to females:

In order to compare the male investment in mating plugs, Plugin and TG3 levels were also quantified from a subset of recently-mated females sampled from the four treatment groups in which 200 virgin females from the KIL or Mopti strains were combined with 200 virgin KIL, Mopti, or heterotic males overnight. Mopti females that were mated to their own males had significantly higher levels of TG3 in the tip of their abdomen than KIL females mated to their own males (Kruskal-Wallis: *χ*^2^ = 9.81, df = 3, *n* = 143, *P* = 0.020; Dunn’s pairwise comparison: *Z* = 3.11, *n* = 72, *P* < 0.011) (Figure 1B). TG3 levels in Mopti and KIL females mated with hybrid males were intermediate and did not differ significantly from the previous two groups (Dunn’s comparisons: *P* > 0.409 in all cases). There were no significant differences in the amount of *Plugin* measured from KIL and Mopti females mated with either type of males (Kruskal-Wallis: *χ*^2^ = 2.29, df = 3, *n* = 122, *P* = 0.515) (Figure 1B).

#### Insemination rates, sperm activity, and numbers:

Another subset of recently-mated females from the four treatment groups were dissected after 48 hr, in order to estimate the rate of female insemination for each experimental group and compare the activity and amount of the sperm contained in their spermathecae. Insemination rates were 93.75% in KIL males with KIL females, 97.92% in Mopti males mated with Mopti females, and 95.83% and 97.92% in hybrid males mated with KIL and Mopti females, respectively, and did not differ significantly among experimental groups (Chi-square of association: *χ*^2^ = 1.63, df = 3, *n* = 192, *P* = 0.652).

**Figure 2:**
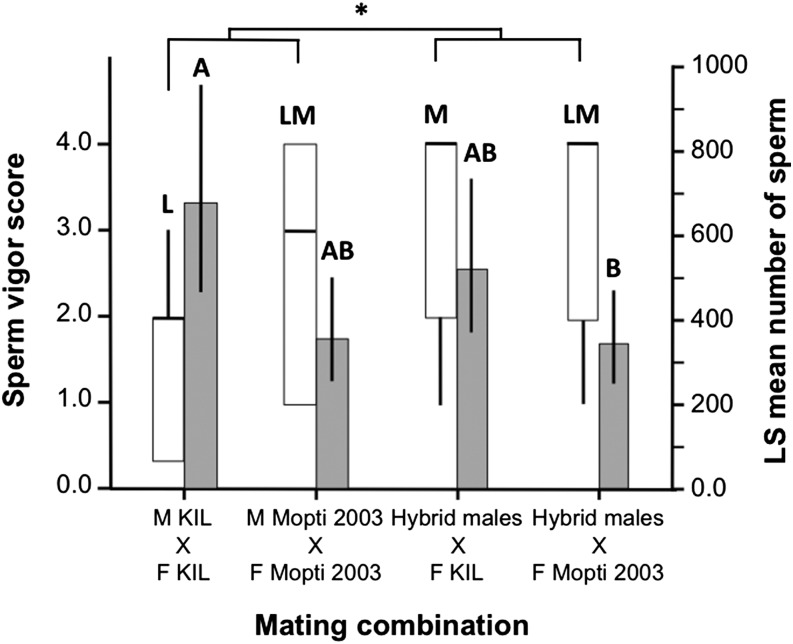
Level of activity and amount of sperm transferred to females in large-group mating cage experiments. Sperm vigor (white bars) and sperm number (gray bars) were compared in KIL and Mopti females mated to KIL and Mopti males or hybrid males. Boxplots indicate medians and first and third quartiles and whiskers are the same quartiles ±(1.5 × interquartile range). Barplots show means (±95% CI). Bars sharing a letter are nonsignificantly different, those with different letters significantly different.

The level of sperm activity measured inside freshly-dissected spermathecae via phase-contrast video microscopy differed significantly among experimental treatment (Kruskal-Wallis: *χ*^2^ = 8.07, df = 3, *n* = 109, *P* = 0.048). Sperm movement was significantly lower in the spermathecae of KIL females mated with KIL males than in those mated with hybrid males (Dunn’s comparisons: *Z* = 2.65, *n* = 50, *P* = 0.048) (Figure 2). Although none of the other pairwise treatment comparisons were significant (*P* > 0.136 in all cases), overall, females mated with hybrid males had more active sperm in their spermathecae than females from other groups (Mann-Whitney: *Z* = 2.0, df = 1, *n* = 107, *P* = 0.045) (Figure 2).

There was no overall statistical difference in the body size of KIL and Mopti females across treatment groups (*T*-test: *T* = –0.52, *n* = 286, *P* = 0.607) (Table 1). However, the mean size of the spermathecae was 0.049 mm (95% CI, 0.047–0.050) in KIL females and 0.046 mm (95% CI, 0.045–0.048) in Mopti females, a small but significant difference between the two strains (*T*-test: *T* = –2.23, *n* = 191, *P* = 0.027) (Table 1). There was no correlation between spermathecae size and sperm vigor in any of the treatment groups (Spearman: *ρ* > 0.147 and *P* > 0.314 in all cases).

**Table 1:** Mean female body size (measured as wing length), spermathecae size, estimated sperm number, and number of eggs and larvae in large-group mating cages for the four experimental mating combinations

**Variable**	**M KIL × F KIL**	**M Mopti × F Mopti**	**Supermales × F KIL**	**Supermales × F Mopti**
Female Wing Length (mm)	2.92 (2.89–2.95)	2.93 (2.90–2.96)	2.93 (2.90–2.95)	2.93 (2.90–2.96)
	*73*	*73*	*70*	*70*
Spermathecae Size (mm^2^)	0.048 (0.046–0.050)	0.046 (0.044–0.047)	0.049 (0.047–0.051)	0.047 (0.045–0.049)
	*49*	*49*	*47*	*46*
Sperm Number*^a^*	669.6 (467.9–958.1)	356 (255.5–498.08)	521.9 (370.8–734.3)	344.0 (248.1–477.0)
	*20*	*23*	*22*	*24*
Eggs per Female	70.2 (57.7–82.7)	64.0 (49.5–78.5)	64.6 (50.1–79.1)	66.2 (52.1–80.3)
	*23*	*17*	*17*	*18*
Larvae per Female	39.4 (29.6–49.1)	35.9 (24.5–47.2)	39.6 (28.3–51.0)	33.9 (22.9–45.0)
	*23*	*17*	*17*	*18*

Ninety-five percent confidence intervals are in brackets and sample sizes in italics.

a Antilog values of the least squares-mean log-transformed sperm number estimates are presented here.

The sperm content (log-transformed) of the spermathecae quantified by qPCR differed significantly among experimental groups and replicates (two-way ANOVA: replicate: *F*_1,84_ = 56.5, *P* < 0.001; treatment: *F*_3,84_ = 3.40, *P* = 0.022). The spermathecae of KIL females mated to KIL males were found to contain significantly more sperm than those of Mopti females inseminated by hybrid males [Tukey test on least squares (LS) means: *P* < 0.05]. KIL females mated to hybrid males and Mopti females mated to Mopti males had intermediate and amounts of sperm in their spermathecae and did not differ statistically from other groups (*P* > 0.05 in both cases) (Figure 2). Overall, the spermathecae of KIL females contained more sperm than that of Mopti 2003 females (two-way ANOVA: replicate: *F*_1,86_ = 57.1, *P* < 0.001; female strain: *F*_1,86_ = 9.3, *P* = 0.003). In KIL females sperm numbers tended to correlate with spermatheca size (Pearson correlation: *r* = 0.268 and *P* = 0.065) but not in Mopti females (Pearson correlation: *r* < –0.233 and *P* = 0.116). Within experimental treatments, sperm number positively correlated with spermatheca size in Kil females mated with hybrid males (*r* = 0.464, *P* = 0.022) while all other groups were nonsignificant.

#### Female and male reproductive success:

There was no significant difference in the mean number of eggs (ANOVA: *F*_3,71_ = 0.18, *P* = 0.910) and larvae (ANOVA: *F*_3,71_ = 0.26, *P* = 0.855) produced by females of the four treatment groups.

### Individual male mating experiments

#### Insemination rates, sperm transferred, and male fertility:

**Figure 3:**
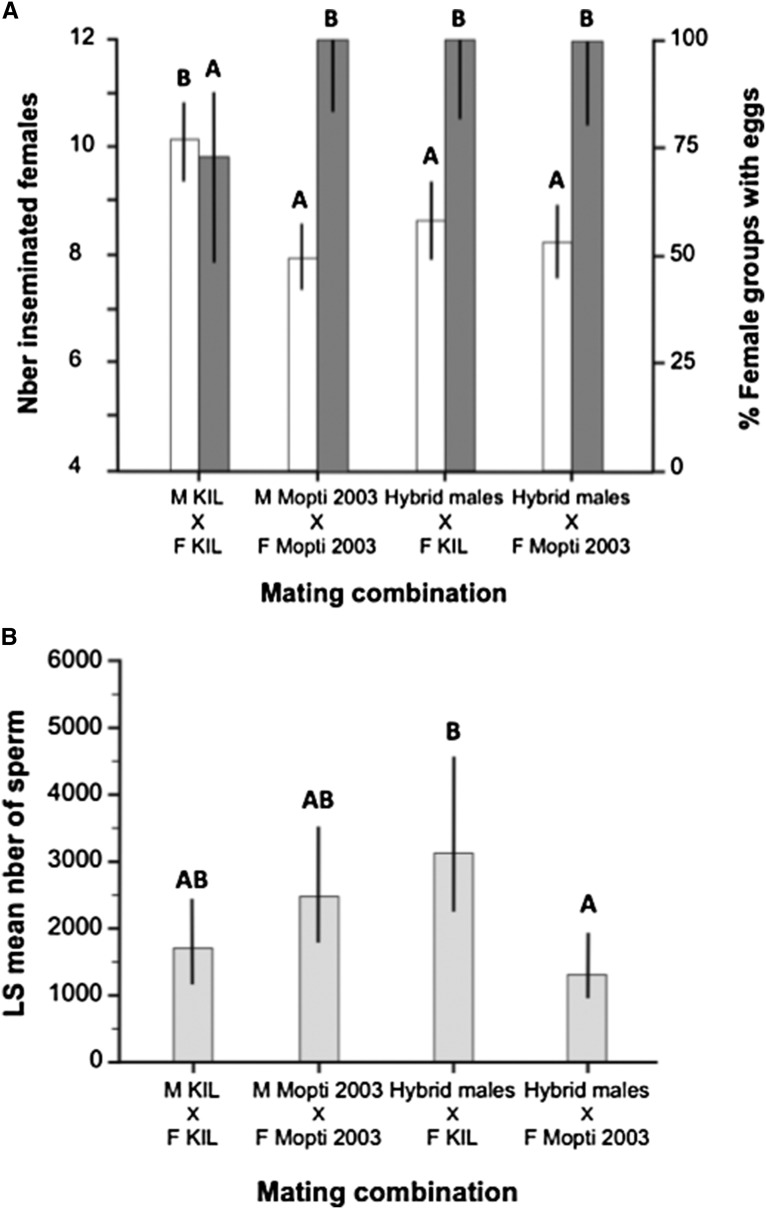
Insemination rate, proportion of female groups with eggs, and mean number of sperm transferred to females in individual-male mating experiments. (A) The number of females inseminated by a single male over three consecutive nights (white bars) and proportion of 20 females groups that did not produce eggs after a blood meal. (B) Qualitative comparison of the mean number of sperm transferred to KIL and Mopti females by individual KIL and Mopti males or hybrid males. Barplots show the group means (±95% CI). Bars sharing a letter are nonsignificantly different, those with different letters significantly different.

Individual males of the old KIL strain inseminated on average half of the 20 virgin females they were kept with for three consecutive nights, which was approximately 20% more than males in the other three treatment groups (ANOVA: *F*_3,62_ = 7.89, *P* < 0.001; Tukey: *P* < 0.017 in all cases) (Figure 3A). However, only 73.33% of female groups mated with individual KIL males subsequently laid eggs suggesting that ∼1/4 of KIL males were sterile and did not induce egg-production in females despite inseminating them (Figure 3A). This contrasted with the KIL and Mopti female groups mated with Mopti or hybrid males, which all produced eggs and larvae (Chi-square: *χ*^2^ = 14.48, df = 3, *n* = 66, *P* = 0.002).

The effect of experimental treatment on the mean amount of sperm transferred per insemination (log-transformed) was tested using an ANOVA with male/cage effects nested within treatment. This analysis revealed quantitative differences between treatment groups but no significant individual male effect (nested ANOVA: Treatment: *F*_3,148_ = 4.29, *P* = 0.006; Male/Cage: *F*_16,148_, *P* = 0.559). The spermathecae of KIL females mated with hybrid males contained significantly more sperm than those of Mopti females inseminated by hybrid males (Tukey test on LS means: *P* < 0.05). KIL and Mopti females mated to males of their own strain contained intermediate amounts of sperm (*P* > 0.05 in both cases) (Figure 3B).

**Figure 4:**
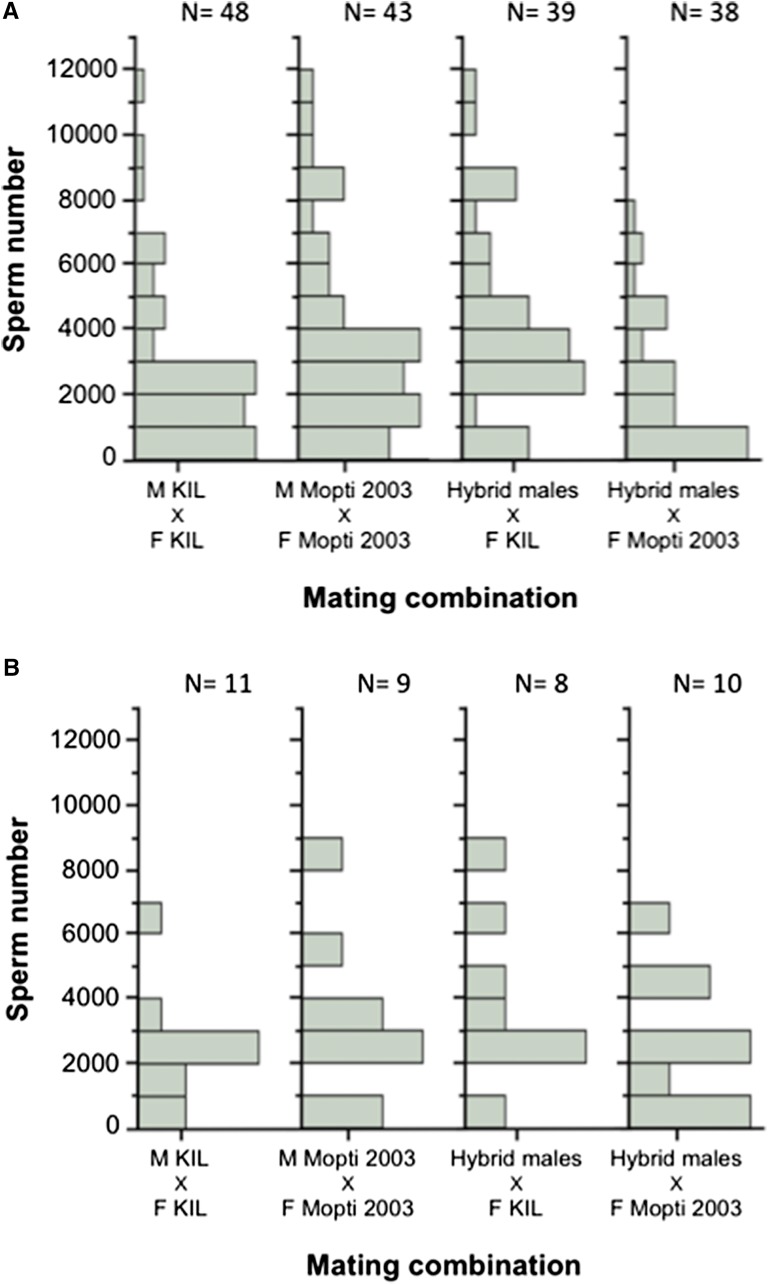
Distribution of sperm numbers recovered from individual female spermathecae. (A) Comparison of sperm amounts transferred to KIL and Mopti females by individual KIL and Mopti males or hybrid males. (B) Distribution of sperm amounts transferred to female in four individual males representative of the four experimental treatment groups and that inseminated respectively 11, 9, 8, and 10 females over three nights.

The number of sperm found in the female spermathecae in individual-male mating experiments varied widely within experimental groups and ranged from 11.2 to 11,799.5 (Figure 4A). This variation was not due to differences between males (see nested analysis above) but rather caused by large differences in the amount of sperm that males transferred to different females (Figure 4B).

#### Female reproductive success:

**Figure 5:**
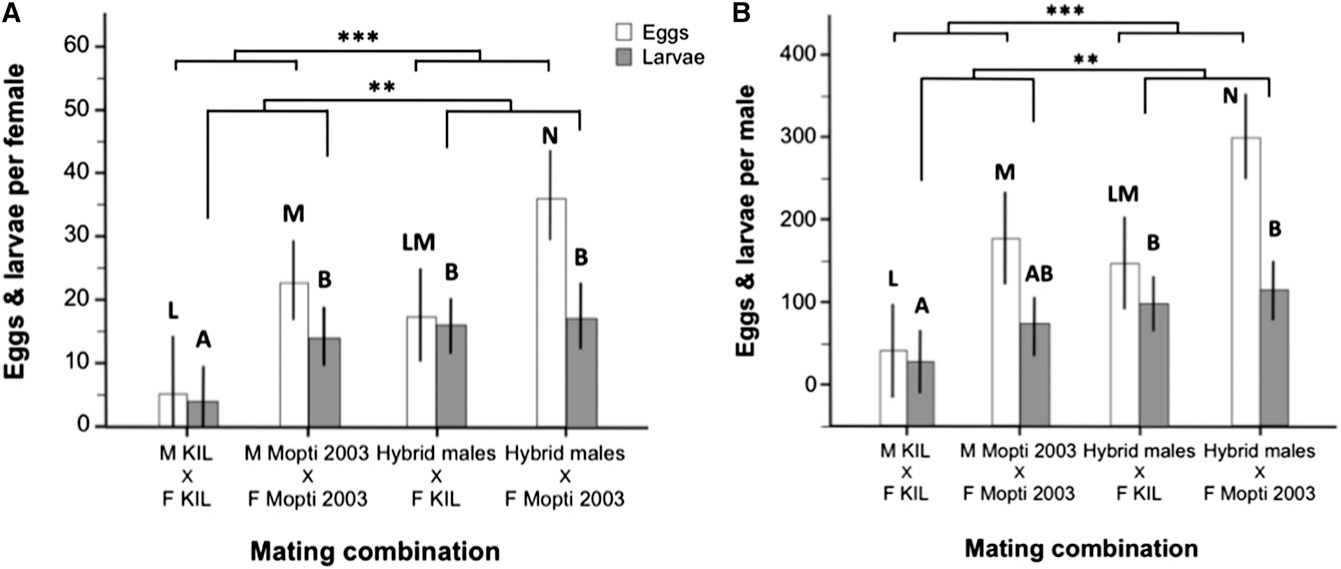
Mean female and individual male reproductive success in individual-male mating experiments. (A) Mean number of eggs (white bars) and larvae (gray bars) produced per mated females for individual male mating cage that produced eggs and larvae in the four treatment groups. (B) Mean number of eggs and larvae calculated per individual male for all mating cages including nonmating ones. Barplots show the group means (±95% CI). Bars sharing a letter are nonsignificantly different, those with different letters significantly different.

There were large and significant differences in the mean number of eggs produced per mated female per individual male mating cages (excluding nonlaying female groups) among the four treatment groups (ANOVA: *F*_3,58_ = 10.51, *P* < 0.001) (Figure 5A). Hybrid males, induced a 3.3-fold and a 1.6-fold increase in the number of eggs developed by KIL and Mopti females compared to females mated to their own males (Tukey: *P* = 0.160 and *P* = 0.040) and Mopti females mated with hybrid males laid significantly more than any other group (*P* < 0.040 in all cases) (Figure 5A). Overall, females mated to hybrid males produced significantly more eggs than those mated to inbred males while Mopti females laid significantly more eggs than KIL ones (two-way ANOVA: male quality: *F*_1,59_ = –3.48, *P* < 0.001; female strain: *F*_1,59_ = 23.04, *P* < 0.001). However, the eggs produced by KIL females were more likely to hatch [median and quartiles: 74.6% (65.0–81.9)] compared to those laid by Mopti females [median and quartiles: 62.5% (29.5–72.5)] (Mann-Whitney: *n* = 57, *Z* = 2.59, *P* = 0.009).

KIL and Mopti females mated to hybrid males produced 4.2 and 1.2 times as many larvae than those mated with males of their own strain (ANOVA: *F*_3,42_ = 5.04, *P* = 0.005) (Figure 5A). KIL females mated to their own males produced fewer larvae than any other groups (*P* < 0.044 in all cases) but there were no statistical differences among the other three treatment groups (*P* > 0.801 in all cases). However, females mated to hybrid males produced significantly more larvae than those mated to inbred males (two-way ANOVA: male quality: *F*_1,58_ = 7.71, *P* < 0.001; female strain: *F*_1,58_ = 2.75, *P* = 0.103) (Figure 5A and Table 2).

**Table 2:** Mean female and male body size (measured as wing length), estimated sperm number, and average number of eggs and larvae produced per mated female per individual-male mating cages for each treatment group

**Variable**	**M KIL × F KIL**	**M Mopti × F Mopti**	**Supermales × F KIL**	**Supermales × F Mopti**
Female Wing Length (mm)	2.93 (2.92–2.94)	2.95 (2.94–2.96)	2.92 (2.91–2.93)	2.94 (2.93–2.95)
	*264*	*318*	*253*	*287*
Male Wing Length (mm)	2.94 (2.91–2.97)	2.94 (2.91–2.97)	2.94 (2.91–2.97)	2.93 (2.90–2.96)
	*15*	*19*	*15*	*17*
Sperm Number*^a^*	1742.3 (1264.9–2400.0)	2496.1 (1779.1–3502.0)	3170.9 (2218.1–4532.9)	1371.7 (953.2–1974.1)
	*48*	*43*	*39*	*38*
Mean Eggs per Mated Female	5.25 (–3.53–14.04)	22.63 (15.95–29.32)	17.49 (9.97–25.01)	35.91 (28.84–42.98)
	*11*	*19*	*15*	*17*
Mean Larvae per Mated Female	3.10 (–2.79–8.98)	9.42 (4.81–14.1)	11.80 (6.76–16.84)	14.16 (9.42–18.89)
	*11*	*18*	*15*	*17*
Mean Eggs per Male	39.4 (–19.3–98.1)	174.7 (122.5–226.9)	145.4 (86.7–204.1)	299.4 (244.3–354.6)
	*15*	*19*	*15*	*17*
Mean Larvae per Male	23.8 (–13.51–66.1)	71.6 (38.4–104.7)	96.7 (59.4–134.0)	111.1 (59.4–134.0)
	*15*	*19*	*15*	*17*

Ninety-five percent confidence intervals are in brackets and sample sizes in italics.

a Antilog values of the least squares-mean log-transformed sperm number estimates are presented here.

#### Male reproductive success:

The reproductive success of individual males was also compared among experimental groups (ANOVA: *F*_3,62_ = 14.17, *P* < 0.001) (Figure 5B). KIL males, despite their higher insemination rates, fathered 3.7 times fewer eggs than hybrid males mated to KIL females (*P* = 0.062) and Mopti males 1.7 times fewer than hybrid males mated to Mopti females (*P* < 0.009) (Figure 5B and Table 2). Hybrid males mated to Mopti females had the highest reproductive success in terms of eggs produced than any other groups (Tukey: *P* < 0.009 in all cases). Overall, hybrid males induced the production of significantly more eggs than inbred males and Mopti females laid significantly more eggs than KIL ones (two-way ANOVA: male quality: *F*_1,63_ = 17.43, *P* < 0.001; female strain: *F*_1,63_ = 26.74, *P* < 0.001)(Figure 5B and Table 2).

Hybrid males also fathered 4.1 and 1.6 times more larvae than KIL or Mopti males mated to their own females (ANOVA: *F*_3,62_ = 4.38, *P* = 0.007, Tukey: *P* < 0.037 and 0.364). Thus, overall, hybrid males fathered significantly more larvae than inbred males (two-way ANOVA: male quality: *F*_1,63_ = 9.47, *P* = 0.003; female strain: *F*_1,63_ = 3.11, *P* = 0.082) (Figure 5B and Table 2).

### Mating plug and sperm transfer in experimental swarms

**Figure 6:**
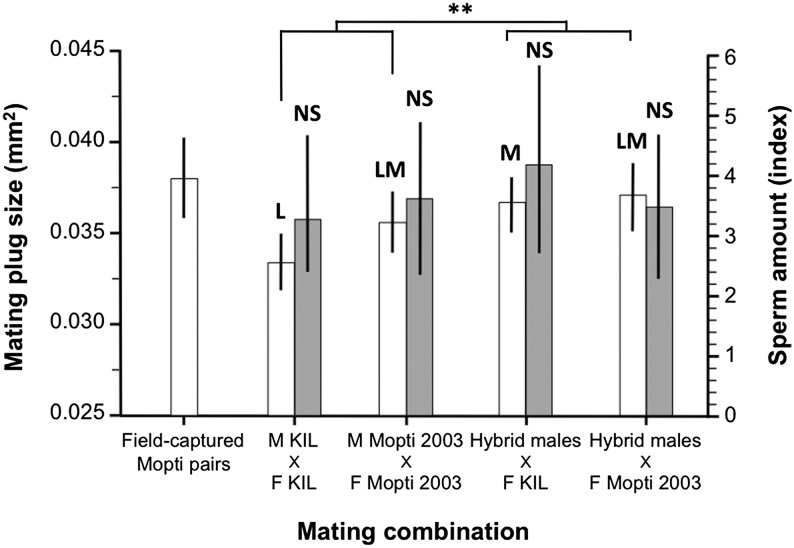
Initial male investment in mating plug and sperm in experimental laboratory swarms. The size of the mating plug (white bars) and the number of sperm (gray bars) recovered in females from couples caught in copula was compared in KIL and Mopti females inseminated by KIL and Mopti males or hybrid males. The size of mating plugs in field-caught couples is also shown for comparisons. Barplots show the group means (±95% CI). Bars sharing a letter are nonsignificantly different, those with different letters significantly different.

The size of the mating plug transferred to females measured from couples captured in copula upon their first mating differed between some of the experimental groups (ANOVA: *F*_3,182_ = 4.67, *P* = 0.004) (Figure 6 and Table 3). Virgin KIL males mated to KIL females transferred significantly smaller mating plugs than hybrid and Mopti males mated to Mopti females (Tukey: *P* < 0.05 in both cases). Hybrid males mated to KIL females had intermediate mating plug size that did not differ significantly from other treatment groups (*P* > 0.051 in all cases). Overall, hybrid males transferred significantly larger mating plugs upon their first mating than inbred males from the parental strains (T-test: *T* = 2.49, *n* = 186, *P* = 0.014). There were no significant among-treatments differences in the amount of sperm (log-transformed) transferred to females by males upon their first mating (ANOVA: replicate: *F*_4,165_ = 8.34, *P* < 0.001; Treatment: *F*_3,165_ = 0.14, *P* = 0.934) (Figure 6 and Table 3). Female (ANOVA: *F*_3,181_ = 1.38, *P* = 0.251) and male (ANOVA: *F*_3,181_ = 0.81, *P* = 0.488) body size did not differ significantly between experimental groups.

**Table 3:** Mean female and male body size (measured as wing length), mating plug size, and sperm transferred in couples captured in copula from experimental swarms of the four treatment groups

**Variable**	**M KIL × F KIL**	**M Mopti × F Mopti**	**Supermales × F KIL**	**Supermales × F Mopti**
Female Wing Length (mm)	3.17 (3.14–3.20)	3.20 (3.17–3.22)	3.17 (3.14–3.19)	3.17 (3.14–3.18)
	*50*	*44*	*47*	*44*
Male Wing Length (mm)	3.01 (2.98–3.05)	2.99 (2.95–3.03)	3.02 (2.99–3.06)	3.02 (2.99–3.06)
	*50*	*44*	*47*	*44*
Mating Plug Size (mm^2^)	0.0334 (0.0318–0.0349)	0.0363 (0.0347–0.0379)	0.0362 (0.0346–0.0378)	0.0373 (0.0357–0.0389)
	*50*	*44*	*47*	*45*
Sperm Amount (Index)*^a^*	4.98 (3.79–6.17)	6.15 (3.65–8.66)	6.11 (4.47–7.75)	4.64 (3.46–5.80)
	*50*	*43*	*45*	*44*

Ninety-five percent confidence intervals are in brackets and sample sizes in italics.

a Here a sperm index was used solely for quantitative comparison purposes due to methodological limitations (see methods)

The size of the mating plug of inbred and hybrid males was also compared to that of females from couples captured in copula in field Mopti swarms (ANOVA: *F*_3,212_ = 4.15, *P* = 0.003). There was no significant size difference between the plugs produced by males from natural Mopti swarms and from laboratory swarms of Mopti males and hybrid males (Tukey: *P* > 0.673 in all cases). However, KIL females inseminated by KIL males had significantly smaller mating plugs in their atrium than field-caught females (*P* = 0.005).

### Male survival experiments

**Figure 7:**
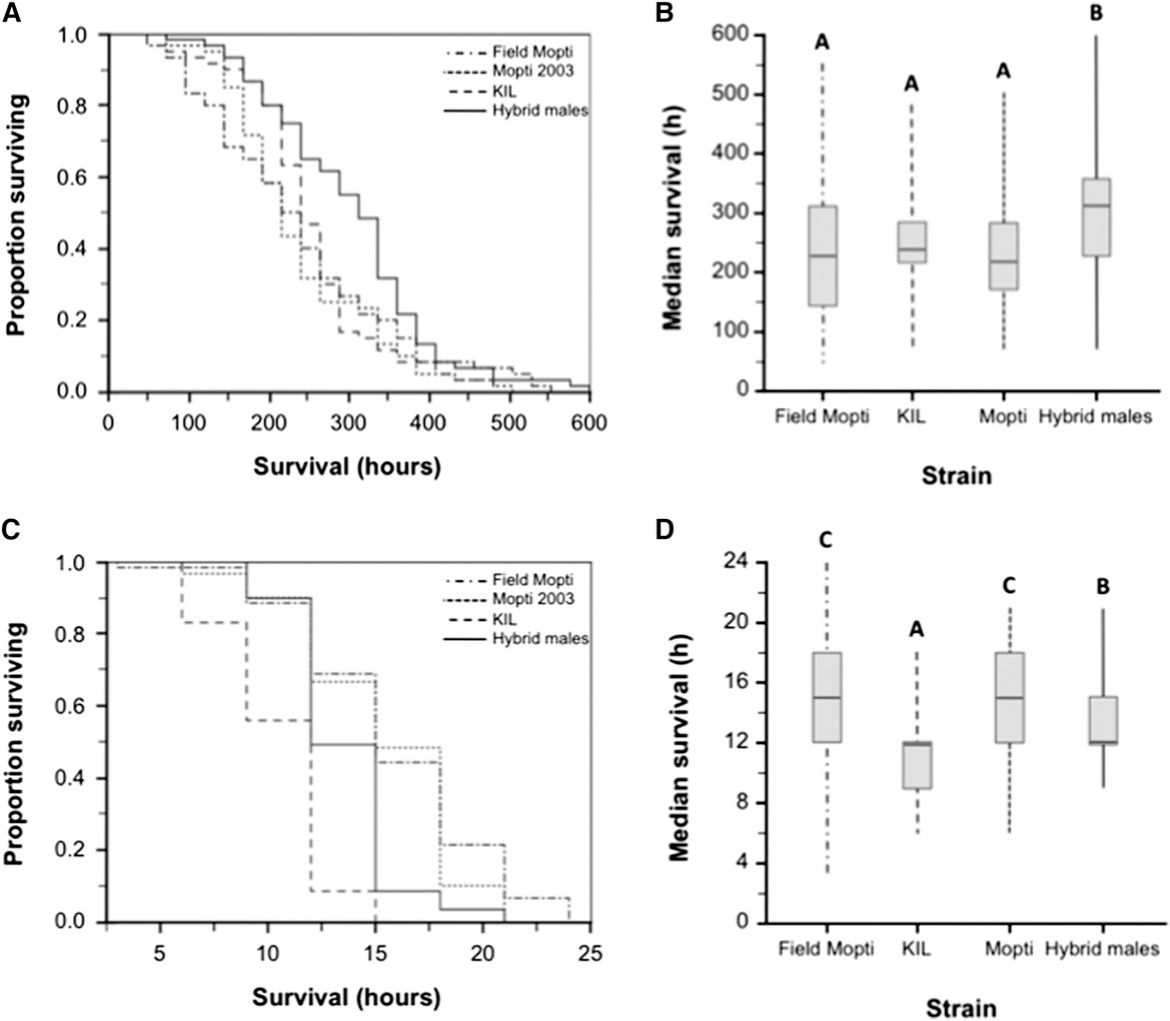
Male survival under standard insectary conditions and intense desiccation. (A, B) Survival curves and median survival in virgin male progeny from field-caught females, males of the KIL and Mopti strains and in heterotic males kept under standard insectary conditions. (C, D) Survival curves and median survival of the four male treatment groups under intense desiccation. Boxplots indicate medians and first and third quartiles and whiskers are the same quartiles ±(1.5 × interquartile range). Bars sharing a letter are nonsignificantly different, those with different letters significantly different.

Overall, hybrid males significantly outlived males from the inbred KIL and Mopti parental strains as well as the male progeny of females captured in the field (Figure 7 and Table 4). The survival of hybrid males and other males strongly interacted with the conditions to which they were exposed and there was no significant effect of male body size on survival (Table 4). Under standard laboratory conditions, hybrid males survived significantly longer than any of the other males, including the male progeny of field-caught females (Kaplan-Meier Log-rank tests: *n* = 240, *P* < 0.003 in all cases). However, under intense desiccation stress, hybrid males survived significantly less than the Mopti laboratory strain and the male progeny from field-caught Mopti females (Kaplan-Meier Log-rank tests: *n* = 239, *P* < 0.001 in both cases) (Figure 7). The Mopti colony and field male progeny originated from the same locality in Mali West Africa (see *Materials and Methods*) and did not differ significantly in survival under either environmental condition (Log-rank tests: *P* > 0.323 in both comparisons). Under standard insectary conditions, the survival of inbred KIL males did not differ significantly from that of Mopti males or the male progeny of field females (*P* > 0.428 in both cases). However, KIL males died faster than any other category of males under intense desiccation stress (*P* < 0.001 in all cases) (Figure 7 and Table 4).

**Table 4:** Multivariate Cox’s proportional hazard analysis of the effects of male treatment group, environmental conditions, and male body size (measured as wing length) on male survival

**Variable**	**df**	**Likelihood-Ratio**	***P*-Value**
Male Group	3	3.5053	< 0.001
Environmental Conditions	1	15.7959	< 0.001
Male Group × Environmental Conditions	1	134.8788	< 0.001
Male Wing Length	1	39.2178	0.262 ns

## Discussion

Despite its potential importance for sterile mosquito releases, this is the first detailed study testing the use of heterosis to detect and counteract the negative impacts of inbreeding on the fitness of laboratory-reared male *An. coluzzii* mosquitoes. In addition to negatively affecting sperm size as reported previously (Baeshen *et al.* 2014), inbreeding was found to negatively impact a variety of male reproductive traits crucial to their reproductive success. These traits included sperm vigor and the size of mating plugs transferred to females. In the most extreme cases, inbreeding led to complete sterility of some males. As a result of these processes, inbreeding strongly negatively impacted the fecundity of females mated to inbred males hence male reproductive success. Finally, inbreeding strongly negatively affected the longevity of inbred males under laboratory conditions.

In all of the above-mentioned traits, heterosis was observed in hybrid males compared to inbred males, thereby highlighting the impact of inbreeding and the potential benefit of producing vigorous hybrid males with improved phenotypic quality. However, in a number of other reproductive traits, the phenotypes of such hybrids were intermediate to those of the inbred parental strains, and therefore followed additive genetic patterns rather than heterotic ones. This suggested widespread adaptations to laboratory rearing in males from the older inbred strain in traits such as the production of plug proteins, mating plug size, sperm transfer, and the rate of female insemination. Additionally, possible adaptive changes resulting in higher sperm uptake such as an increase in the size of spermathecae were also observed in females. Predictably, the >35-year-old KIL strain was generally more affected by inbreeding and selection for laboratory conditions than the 8-year-old Mopti strain and this pattern was confirmed across all studied fitness traits.

### Adaptations to mating in the laboratory in inbred strains

In a previous study, male *An. coluzzii* from laboratory strains were shown to have evolved larger testes and smaller accessory glands over time (Baeshen *et al.* 2014). Here, males from the 30-year-old KIL strain were also found to produce higher precopulatory levels of TG3 than Mopti males, heterotic males and ’field males’. In anophelines, TG3 forms the matrix of the mating plug and is crucial for cross-linking other plug compounds as well as inducing sperm uptake by females (Rogers *et al.* 2009; Le *et al.* 2013). When mating overnight under crowded conditions, KIL males were found to transfer lesser amounts of TG3 but more sperm to females than Mopti males, while hybrid males transferred intermediate amounts of TG3 and sperm. These differences therefore mirrored those previously observed in the size of testes and accessory glands in relation to the time since colonization (Baeshen *et al.* 2014) and suggest an adaptive change in the balance of sperm to plug investment in response to laboratory breeding. It is noteworthy that the same patterns were not observed in the *Plugin* protein. This protein plays a constitutive role in the outer layer of plugs and is not known to regulate female fertility and might therefore not be under the same selection pressures as TG3.

Individual male mating experiments revealed that KIL males were capable of inseminating more females per night than Mopti and hybrid males thereby providing a functional explanation for the adaptive changes observed changes in their reproductive organ size and in their sperm and TG3 investments. Finally, in experimental swarms, virgin KIL males transferred smaller plugs but equal amounts of sperm to females than Mopti and hybrid males, which further supports the idea that the change in plug investment is, at least in part, the result of adaptation to mating in the laboratory (but see *Reproductive traits affected by inbreeding and heterosis*).

Taken together, these results suggest that inbred KIL males have undergone profound changes to adapt to mating conditions in the laboratory where large numbers of males and females emerge simultaneously in confined cages. Such conditions effectively create an artificial mating system that contrasts with the elaborate and sequential mate selection processes suspected to take place in natural swarms (Diabate and Tripet 2015). In laboratory cages males can maximize their reproductive success by quickly inseminating the highest possible number of virgin females until all available females are mated (Baeshen *et al.* 2014). Males of the old KIL laboratory strain seem to have adapted to these conditions by having larger testes, therefore being capable of inseminating more females then other males, albeit transmitting comparatively smaller mating plugs to females than other males. The higher amounts of TG3 found in the KIL males accessory glands suggest that, despite their smaller size, these plugs might contain a comparatively higher concentration of TG3 relative to other compounds. Given the known importance of TG3 for sperm uptake and storage by females (Rogers *et al.* 2009), these results suggest that the composition of the plug itself might have changed in response to mating in under laboratory conditions.

Despite the general knowledge that laboratory strains do become better adapted to insectary rearing overtime, how these changes affect the male mating phenotype beyond simple insemination rates has seldom been reported. However, adaptation for earlier mating under laboratory conditions was also observed in *An. arabiensis* strains reared in the laboratory in preparation of sterile mosquito releases programs in the Soudan (Oliva *et al.* 2011). In this case, male sexual maturation characterized by the 180° rotation of their genitalia, occurred much faster in laboratory males of this species than in their wild counterparts, allowing mating to take place sooner after emergence in the laboratory (Oliva *et al.* 2011). Evidence of adaptations to laboratory breeding has also been observed in fruit fly species reared for sterile male release programs, in which changes such as shortened preoviposition period, increased early fecundity and survival in long-established strains and are considered favorable (Vargas and Carey 1989; Meats *et al.* 2004; Hernandez *et al.* 2009; Gilchrist and Meats 2014). However, concurrent changes in mating behavior that negatively impact male mating competitiveness are also well known, prompting complex schemes attempting to regain mating competitiveness without losing the benefits of higher productivity (McInnis *et al.* 1996, 2002; Shelly 2001; Rull and Barreda-Landa 2007; Rull *et al.* 2012; Gilchrist and Meats 2014).

### Reproductive traits affected by inbreeding and heterosis

Here, prior to assessing heterosis in reproductive traits of inbred and supermales, we determined the genetic diversity of the parental laboratory strains. Genetic diversity was found to be ∼20% lower in the >8-year-old Mopti 2003 laboratory colony originating from Mali than in a field Mopti population from neighboring West Burkina Faso. Previous studies have shown that genetic differentiation within the Mopti form of *An. coluzzii* and across such small geographical scales is generally low, suggesting that these populations may belong to a large panmictic unit occupying the tropical dry and shrubland ecological zones (Slotman *et al.* 2007; Tripet *et al.* 2005a). Consequently, the decrease in genetic diversity observed between the field and laboratory populations is likely to reflect colonization rather than demographic peculiarities between their respective field populations of origin. Genetic diversity was also found to ∼3% lower in the >35-year-old KIL strain than in the Mopti 2003 strains and, although we do not have data on this strain’s original field population, its low genetic diversity supports the idea that mosquito colonies continuously lose genetic variation over time thus making them prone to the negative fitness effects of inbreeding. Albeit correlations between individual multilocus heterozygosities and fitness are generally weak (Chapman *et al.* 2009) and depend on the number of markers used (Balloux *et al.* 2004), mean heterozygosity within populations has been shown to correlate with the negative inbreeding effects in a number of natural and experimental populations (Reed and Frankham 2003; Markert *et al.* 2010).

In this study, the effects of inbreeding and heterosis on male reproduction were detected at many levels. Large-group mating experiments revealed that the sperm of KIL males was less active in the spermathecae than that of Mopti males and supermales. Here, sperm activity in the spermathecae was used as a proxy for the proportion of live sperm produced by males—an important determinant of fertility negatively affected by inbreeding (Fitzpatrick and Evans 2009; Losdat *et al.* 2014). Spermathecal sperm activity can also reflect differences in sperm motility or vigor, another determinant of fertility known negatively affected by reduced heterozygosity (Fitzpatrick and Evans 2009; Losdat *et al.* 2014). The lower sperm activity of KIL males did not lead to significantly reduced female fecundity in the large-group mating experiments. This may be because of the high mosquito density used to achieve the high insemination rates needed in these experiments. Under such conditions, the KIL males that successfully inseminated females transferred a significantly larger amount of sperm to females than the Mopti and supermales males, and this may have compensated for their overall lower sperm activity.

The experimental design of individual-male mating experiments enabled us to detect much subtler differences in male phenotypic quality by tracking the male mating performance over three nights and removing the possible effect of female mate choice on male performance by randomly picking males. KIL males inseminated an average 3.37 females per night, significantly more than the 2.75 females inseminated across other types of males. However, in contrast to what was found in the overnight large-group mating cage experiments, KIL males did not transfer significantly larger amounts of sperm per insemination over three nights, indicating that, despite their larger testes, they did not replenish their sperm reserves at a faster rate that Mopti and supermales. Previous studies have claimed that *An. gambiae* s.l. males could inseminate up to five females per night (Giglioli and Mason 1966). Here, the 72-hr span of our mating experiments prevents direct comparisons of nightly insemination rates with previous studies, but the lower overall lower mating rate suggests that if virgin males were indeed capable of inseminated as many as five females in the first night they would have slowed down in subsequent nights.

A surprising discovery was the high rate of sterility uncovered in KIL males. One in four KIL males failed to induce the production of any eggs among any of the groups of females that they inseminated over three nights. KIL male sterility could possibly be explained by their lower sperm activity. Unfortunately, we were unable to match the sperm activity and fertility of individual males in this experiment as the quantification of sperm transferred to female required storage of whole-bodied females under standard conditions. In a previous study, the length of KIL sperm was found to be strongly reduced (Baeshen *et al.* 2014). Therefore, sterility in KIL males could also be due to the particularly high proportion of small sperm in their ejaculate or to unknown sperm defects caused by inbreeding that can affect fecundity (Baeshen *et al.* 2014). Additionally, in experimental-swarm mating experiments, a small but significant overall decrease in the size of the mating plugs produced by KIL males compared to those of supermales was recorded. Thus, despite the results of previous studies and our large-group mating experiments which suggest that male investment in the mating plug and its constituents has undergone adaptive changes in response to mating in the laboratory, we cannot rule out the possibility that inbreeding could have affected the composition of the mating plug, with negative carry-over effects on fertility. Recent studies have highlighted the role of male-produced TG3 and 20-hydroxy-ecdyzone for sperm uptake in the spermathecae and for inducing the production of the *MISO* protein and *HPX15* peroxidase—two molecules that play a critical role for sperm storage and oogenesis (Rogers *et al.* 2009; Baldini *et al.* 2013; Shaw *et al.* 2014). Therefore changes in plug composition brought about by inbreeding could interfere with these processes and possibly cause male sterility.

Unsurprisingly, the positive impact of heterosis was particularly evident when measuring the end result of all reproductive processes potentially affected by inbreeding, *i.e.*, total male reproductive success. When considering only fertile males in the individual-male experiments, hybrid males induced a ∼two-fold increase in the number of eggs and larvae produced by females compared to KIL and Mopti males. When total male fecundity was considered, which included both sterile and fertile males, hybrid males were again found to increase the number of eggs and larvae produced by females ∼two-fold compared to inbred males. Despite the high rate of sterility in KIL males, the mean number of larvae fathered by KIL males was not as severely affected as expected. This was due to KIL males inseminating more females than other males and the higher overall hatching rates of the eggs laid by KIL females compared to those from Mopti females. This difference could again result from adaptations to laboratory conditions (see *Adaptations to mating in the laboratory in inbred strains*) affecting the development of eggs. KIL females might also have progressively adapted to the increasingly poor sperm quality of their inbred males by developing a larger spermathecae in order take up as much sperm as possible upon mating. This would in turn maximize their rates of egg fertilization.

Within all treatment groups, the quantification of sperm numbers in the individual-male mating experiment revealed an astounding variation in spermathecal sperm content. In KIL females mated with KIL males, for example, sperm estimates ranged from 50 to 11,799, equivalent to a ∼200-fold difference. Understandably, this experiment was specifically designed to push male insemination ability and sperm production to their limits. However, high variation was also found in the large-group overnight mating and swarm experiments where we recorded ∼20- to 50-fold differences between the smallest and largest estimates of sperm content. This variation highlights the strong selection pressures that female monandry and high fecundity impose on male sperm and plug production.

### Inbreeding, heterosis, and longevity

While male mating competitiveness and male fecundity are important for male release programs aiming to achieve population suppression and replacement, male survival is a fitness parameter that is crucial to all male release strategies (Lance and McInnis 2005). Field studies of male mating success in natural *An. coluzzii* swarms have shown that the optimum male age for successful insemination of females was 4 days old (Sawadogo *et al.* 2013). Therefore the survival of males released at a younger age can greatly impact their initial mating success and, generally, their lifetime mating success that is crucial to male release programs. Here, under insectary conditions, hybrid males significantly outlived all other types of males, including the older KIL strain, the comparatively younger Mopti strain colonized from a population in the vicinity of Bamako in Mali, and the progeny of field females collected from the same locale. The average intrinsic longevity of hybrid males was increased by 36.8% compared to other males suggesting a strong positive effect of heterosis.

Under the harsh conditions of the desiccation challenge, however, the survival of hybrid males was higher than that of KIL but lower than that of the Mopti colony and the Mopti field progeny. This interaction between male type and environmental conditions and their effect on longevity can be explained by the presence of inversion polymorphisms in the Mopti strain and the Mopti field progeny that confer resistance to drought (Lee *et al.* 2009; Aboagye-Antwi *et al.* 2010). The Mopti chromosomal form of *An. coluzzii* occupies semiarid regions of sub-Saharan Africa where seasonal variations in water availability and ambient humidity are important determinants of mosquito distribution (Toure *et al.* 1994, 1998; Charlwood *et al.* 2000). In such habitats, the relative abundances of the Mopti chromosomal form of *An. coluzzii* and the Savanna and Bamako forms of *An. gambiae* s.s. are strongly associated with seasonal rainfalls thereby underlining the role of chromosomal inversion polymorphisms in conferring resistance to desiccation (Toure *et al.* 1994, 1998). The Mopti chromosomal form, characterized by the *bc* and *u* inversion on chromosome 2R and fixed for the inversion *a* on 2L, is the most resistant to drought and predominates in dry areas and during the dry season (Toure *et al.* 1994, 1998). Therefore the *u* and *bc* inversions that are found in the Mopti strain and the Mopti progeny of field-caught females but absent from the KIL strain could explain the lower survival of the latter strain under desiccation stress. Similarly, the 2L*a* inversion occurs only at low frequency in the KIL strain and is thought to play a role in desiccation (Toure *et al.* 1998; Fouet *et al.* 2012). Hybrid enhanced males were *a fortiori* heterozygous for the inversions karyotypes of their parental Mopti and KIL strains, which therefore led to their intermediate desiccation resistance phenotype. This observation emphasizes the necessity of carefully considering the genetic background of hybrid males and not only simply their vigor in planning release programs.

### Conclusions

The results reported here suggest that, notwithstanding the negative effects of inbreeding, old mosquito strains are adapted to laboratory breeding to an extent previously unsuspected. Overtime, the artificial mating system imposed on laboratory strains has deeply changed the male mating phenotype, going as far as changing their insemination capacity, and imposing changes in the size and composition of the mating plug transferred to females with possible effects on sperm uptake and retention. Inbreeding deeply affected the older strain, resulting in male sterility and a drastic decreased in male and female fecundity. Further studies are required to assess the relative importance of decreased sperm size, sperm activity, and plug size or composition on male reproductive success.

For male release programs to be effective, the negative effects of inbreeding on laboratory-reared mosquitoes need to be counteracted using schemes for reconstituting their genetic diversity and phenotypic quality (Benedict *et al.* 2009). Such schemes typically require regular crossing and backcrossing laboratory strains with the progeny of wild caught individuals, and thus are not always practical to implement (Benedict *et al.* 2009). The demonstration that heterosis can be used for improving male longevity and rescuing key aspects of the male reproductive phenotype, potentially resulting in vastly increased lifetime reproductive success offers a major boost in the feasibility of prospective mosquito release strategies and integrated diseases control programs. Given that the adaptations to mating in the laboratory detected in males of old colonized strains are likely to negatively affect male mating competitiveness of any F_1_ hybrid males when released into natural populations, the heterosis strategy is likely to work best using F_1_ produced from inbred lines created from established but not excessively old laboratory strains. Future studies should aim to directly evaluate the potential benefits of heterosis on male mating competitiveness under semifield and field conditions.

## Supplementary Material

Supporting Information
