# Supplementary material for: Heterosis Increases Fertility, Fecundity, and Survival of Laboratory-Produced F1 Hybrid Males of the Malaria Mosquito Anopheles coluzzii
Source: G3 (Bethesda). 2015 Oct 20;5(12):2693–709. doi: 10.1534/g3.115.021436 (PMC4683642; doi:10.1534/g3.115.021436)
Supplement: Supporting Information [file supp_5_12_2693__index.html]

Heterosis Increases Fertility, Fecundity and Survival of Laboratory-Produced F1 Hybrid Males of the Malaria Mosquito Anopheles coluzzii — Heterosis Increases Fertility, Fecundity, and Survival of Laboratory-Produced F1 Hybrid Males of the Malaria Mosquito Anopheles coluzzii — Supporting Information 

# Heterosis Increases Fertility, Fecundity, and Survival of Laboratory-Produced F1 Hybrid Males of the Malaria Mosquito *Anopheles coluzzii*

## Supporting Information for Ekechukwu *et al.*, 2015

**Files in this Data Supplement:**

- File S1 - Supplementary data file. (.zip, 544 KB)
